# Mucin-Type *O*-Glycosylation in Gastric Carcinogenesis

**DOI:** 10.3390/biom6030033

**Published:** 2016-07-11

**Authors:** Henrique O. Duarte, Daniela Freitas, Catarina Gomes, Joana Gomes, Ana Magalhães, Celso A. Reis

**Affiliations:** 1 i3S—Instituto de Investigação e Inovação em Saúde, Universidade do Porto, Rua Júlio Amaral de Carvalho, 45, Porto 4200-135, Portugal; hduarte@ipatimup.pt (H.O.D.); dfreitas@ipatimup.pt (D.F.); cgomes@ipatimup.pt (C.G.); joanag@ipatimup.pt (J.G.); amagalhaes@ipatimup.pt (A.M.); 2 Institute of Molecular Pathology and Immunology of University of Porto, Ipatimup, Rua Júlio Amaral de Carvalho, 45, Porto 4200-135, Portugal; 3 Instituto de Ciências Biomédicas de Abel Salazar (ICBAS), University of Porto, Rua Jorge Viterbo Ferreira no. 228, Porto 4050-313, Portugal; 4 Medical Faculty, University of Porto, Alameda Prof Hernâni Monteiro, Porto 4200-319, Portugal

**Keywords:** gastric cancer, mucins, *O*-glycosylation, glycosyltransferases, *Helicobacter pylori*, Lewis antigens

## Abstract

Mucin-type *O*-glycosylation plays a crucial role in several physiological and pathological processes of the gastric tissue. Modifications in enzymes responsible for key glycosylation steps and the consequent abnormal biosynthesis and expression of their glycan products constitute well-established molecular hallmarks of disease state. This review addresses the major role played by mucins and associated *O*-glycan structures in *Helicobacter pylori* adhesion to the gastric mucosa and the subsequent establishment of a chronic infection, with concomitant drastic alterations of the gastric epithelium glycophenotype. Furthermore, alterations of mucin expression pattern and glycan signatures occurring in preneoplastic lesions and in gastric carcinoma are also described, as well as their impact throughout the gastric carcinogenesis cascade and in cancer progression. Altogether, mucin-type *O*-glycosylation alterations may represent promising biomarkers with potential screening and prognostic applications, as well as predictors of cancer patients’ response to therapy.

## 1. Introduction

**Figure 1:**
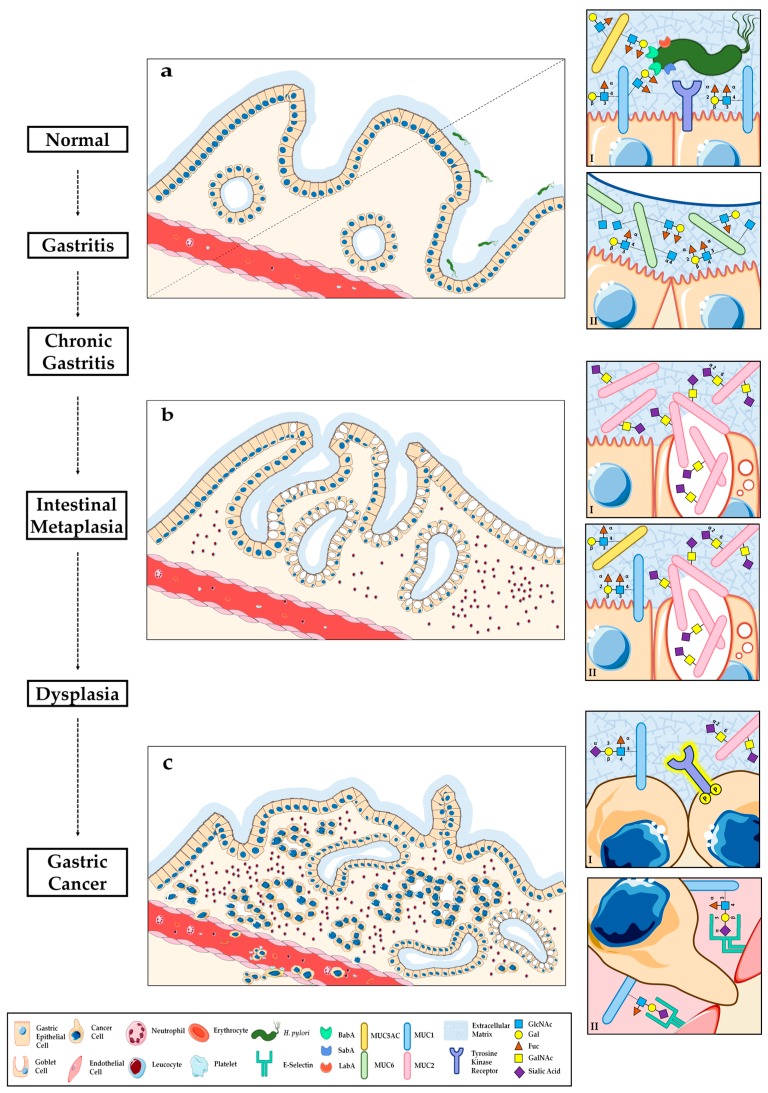
Schematic representation of mucin expression pattern and associated *O*-glycan signatures through the gastric carcinogenesis cascade: (**a**) Normal mucous-secreting gastric epithelium (left) and early stage of *H. pylori* colonization (right); (**aI**) Superficial foveolar epithelial cells expressing membrane-bound MUC1 and secreted MUC5AC associated with type 1 Lewis antigens, which serve as receptors for *H. pylori* BabA-mediated adhesion, promoting infection and leading to gastritis; (**aII**) Glandular epithelial cells expressing the secreted MUC6 associated with type 2 Lewis antigens and the terminal α1,4-GlcNAc natural antibiotic glycan structure; (**b**) Intestinal metaplasia (IM) with goblet cells and inflammatory infiltrate; (**bI**) Complete type IM, with marked secretion of intestinal MUC2 carrying STn and absence of gastric mucins; (**bII**) Incomplete type IM, with co-expression of gastric and intestinal mucins; (**c**) Intestinal type gastric adenocarcinoma with disorganized glandular architecture and inflammatory infiltrate; (**cI**) Gastric cancer cells displaying aberrant surface glycosylation with concomitant activation of TKR-dependent signalling pathways; (**cII**) Sialylated Lewis antigens potentiate the invasive and metastatic capacity of GC cells, by serving as ligands to endothelial selectins during tumour cell extravasation.

Gastric carcinogenesis is a sequential multistep process that encompasses molecular, genetic and histologic alterations, ultimately leading to the development of gastric cancer (GC) (Figure 1) [1]. Intestinal type GC constitutes a complex multifactorial pathway resulting from the interplay of environmental risk factors, infection with *Helicobacter pylori* (*H. pylori*) bacterium, classified as a type I human carcinogen [2], and host genetic susceptibility [1,3].

*H. pylori* persistent infection of the normal gastric mucosa triggers a chronic inflammatory process designated by chronic gastritis. The presence of virulent *H. pylori* strains together with host immune vulnerability can lead to severe mucosal atrophy with focal loss of gland architecture and disease progression [4]. In the atrophic gastric mucosa, the loss of acid-secreting parietal cells elevates gastric pH and promotes the proliferation of anaerobic bacteria, as well as successive alterations. In fact, the development of intestinal metaplasia (IM) originates multiple foci where superficial foveolar cells with neutral mucin expression are gradually replaced by acidic sialomucin-producing cells with an intestinal phenotype [1]. This precancerous lesion is associated with an increased risk of GC development. Dysplasia, which encompasses significant nuclear atypia and tissue architectural distortion, may also appear before the cells acquire the capacity to invade and metastasize [4].

The cellular glycosylation profile is crucial for several physiological and pathological mechanisms. It has been shown that the abnormal expression of enzymes controlling key glycosylation steps, or alterations of their glycan products, have a clear association with GC onset and progression, through their implication in several features of tumour cell biology and behaviour [5]. These glycan alterations occurring during the gastric carcinogenic pathway include increased expression of sialylated terminal structures, as well as aberrant expression of simple-mucin-type carbohydrate antigens. Glycans represent well-established key mediators of different aspects of tumour progression, regulating several processes, such as proliferation, invasion, metastasis and angiogenesis [6].

## 2. Mucin and *O*-Glycosylation in Healthy Gastric Mucosa

Like all other epitheliums, the surface of the human gastrointestinal tract is covered by a viscous layer of mucus, which functions as an essential protective barrier against several sources of physical, chemical and biological stress (Figure 1a) [7]. Mucins, a family of large and heavily glycosylated proteins, represent the main component of this hydrophilic gel-like mixture, and act as key molecules in the maintenance of gastrointestinal homeostasis. Placed at the interface with the gastric lumen, mucin glycoproteins secure the integrity of the underlying layer of epithelial cells, shielding it from the adverse mechanical and biochemical conditions of the stomach external environment, such as the presence of digestive enzymes and the acidic pH [8]. In addition to this protective role, the biochemical properties of mucins greatly influence the levels of gastric epithelium lubrication and tightly regulate processes of selective molecular trafficking. Furthermore, mucins can serve as surface receptors for the binding of several adhesion molecules, mediate the interaction with pathogens, and locally modulate the inflammatory response, all by acting as highly specialized molecular sensors capable of triggering intracellular signalling pathways in response to distinct stimuli from the external microenvironment [9].

Presently, 22 members of the mucin (MUC) protein family have been identified, and can be sub-classified as either membrane-bound (transmembrane) or secreted (gel-forming) [10]. Membrane-associated mucins are involved in the maintenance of epithelial cell polarity and signal transduction by interaction of their cytosolic tail with intracellular proteins. Secreted extracellular mucins are responsible for conferring the viscoelastic properties of the mucous layer lining epithelial tissues [9]. Localized at the apical borders of the epithelial cells that produce them, mucins show a highly specific cell- and tissue-dependent expression pattern [11,12]. In non-pathological conditions, the human gastric mucosa is mainly characterized by the expression of three mucins with a well-defined distribution profile across distinct populations of mucous-secreting cells [13,14,15]. Cells from the surface foveolar epithelium express both the secreted MUC5AC and the membrane-bound MUC1, being the latter also found in the neck gland cells (Figure 1aI). As for the deeper layers of the gastric mucosa, MUC6 is secreted by the neck glandular cells (Figure 1aII). Expression levels of each gastric mucin may display moderate variations among the distinct regions of the stomach [11,12]. A common structural feature of all mucin family members is the existence of a variable number of tandem-repeat domains, also known as mucin domains, characterized by the sequential alignment of highly similar amino acid sequences rich in Proline, Threonine and Serine [16,17]. Due to the elevated content of these amino acid residues, mucin domains suffer extensive *O*-glycosylation, which ultimately defines mucin biochemical properties and function [18]. In fact, mucin oligosaccharides may represent up to 90% of the total molecular weight of the mature glycoprotein [19]. In mucin-type *O*-glycosylation, sugar molecules are individually and sequentially added to the protein backbone by a set of distinct enzymes with glycosyltransferase activity, associated with the membrane of the Golgi apparatus. The first step of this process consists in the transfer of the *N*-Acetylgalactosamine (GalNAc) monosaccharide from a Uridine Diphosphate *N*-Acetylgalactosamine (UDP-GalNAc) sugar donor to the hydroxyl group of either a Serine or a Threonine, and is catalysed by a group of UDP-GalNAc:polypeptide *N*-Acetylgalactosaminyltransferases (ppGalNAc-Ts) [20,21]. Therefore, this enzyme family, which presently comprises 20 distinct members, controls and tightly regulates *O*-glycan site occupancy and density at the mucin tandem-repeat domains, the first level of complexity of mucin-like *O*-glycosylation [22,23]. Despite catalysing the same enzymatic step, each ppGalNAc-T isoform has its own set of kinetic properties and substrate specificity, as well as a unique and tissue-dependent pattern of expression, which ultimately determines enzymatic function and the *O*-glycosylation profile of tissues and organs in normal physiological conditions [24,25]. However, substantial functional overlaps between different isoforms have been previously reported, and may represent cellular compensatory mechanisms regulating mucin-like *O*-glycosylation [26,27]. Despite the expression pattern of certain ppGalNAc-T isoforms being strictly restricted to specific tissues, others show a rather ubiquitous expression across multiple organs. Regarding the human gastric mucosa, the presence, both at the transcript and protein level, of several members of the ppGalNAc-T enzyme family (-T4, -T5, -T6, -T10 and -T12) has previously been reported [20,28,29,30,31]. Moreover, studies addressing ppGalNAc-T immunohistochemical tissue distribution and mucin substrate-depending activity further indicate the existence of a delicate regulatory network that finely tunes ppGalNAc-T-mediated *O*-glycosylation. 

Mucin carbohydrate chains frequently carry terminal structures known as Lewis blood group antigens, which can be classified as type 1 (Le^a^, Le^b^ and sialyl-Le^a^ (SLe^a^)) and type 2 (Le^x^, Le^y^ and sialyl-Le^x^ (SLe^x^)) [32]. Their biosynthesis is mediated by the coordinated action of enzymes with fucosyltransferase (FUT) and sialyltransferase activity showing a cell- and tissue-specific expression pattern, and is further determined by the individual genotype [33]. FUT2 acts primarily by adding a fucose residue to type 1 precursor chains, and, in the human normal gastric mucosa, has its expression restricted to the surface foveolar epithelium, where it co-localizes with MUC5AC associated with type 1 Lewis antigens (Figure 1aI). The expression of FUT1, which catalyses, in turn, the fucosylation of type 2 precursor chains, is mainly observed in the deep glands of the stomach, where it co-localizes with MUC6 associated with type 2 Lewis antigens (Figure 1aII). Expression of FUT3, the enzyme responsible for the difucosylation of type 1 and 2 Lewis antigens, can be detected in both gastric cell populations [34]. Distinct members of the sialyltransferase enzyme family can give rise to sialylated versions of Le^a^ and Le^x^ structures, although the expression of these antigens in the normal gastric epithelium is a rather uncommon event [33].

Additionally, MUC6 *O*-glycan chains carry terminal *N*-Acetylglucosamine residues, which have been reported to have a protective role against pathogen infection and are synthesized by the enzyme α1,4-*N*-Acetylglucosaminyltransferase, specifically produced by glandular mucous cells, where it co-localizes with the expression of MUC6 (Figure 1aII) [35,36,37].

Altogether, it seems that the glycosylation profile of the human normal gastric mucosa is greatly dictated by the finely-tuned co-expression of a set of distinct glycosyltransferases and their preferential gastric mucin substrates, following a tissue- and cell-specific pattern.

## 3. *O-*Glycosylation Alterations during Gastric Carcinogenesis

### 3.1. Helicobacter Pylori Host Glycan Receptors and Infection

The glycan structures expressed on the mucus layer and cells lining the gastric epithelium constitute an important interface for bacterial binding and stomach colonization [38,39]. While for most microbes the stomach constitutes a hostile microenvironment, the carcinogenic bacteria *H. pylori* are well adapted to this niche and chronically colonize the gastric mucosa of more than 50% of the world population. *H. pylori* infection is the main risk factor for GC development and, in agreement, *H. pylori* eradication strategies have been shown to significantly reduce GC risk among patients without advanced preneoplastic changes [40,41,42].

*H. pylori* attachment to the gastric epithelium is critical for infection success by providing access to nutrients, decreasing exposure to the very acidic lumen pH, and promoting the delivery of bacterial virulence factors [39]. Bacterial attachment to the epithelial cells is mediated through host glycan recognition by a set of outer membrane proteins (OMP), belonging to the Hop family that present lectin-like binding properties. The ABO/Le^b^ histo blood group antigens constitute ligands for the Blood group antigen binding adhesin (BabA) (Figure 1aI) [43,44]. A high BabA sequence diversity has been reported among clinical isolates and, according to BabA binding affinities, bacteria can be divided into specialist and generalist strains. The generalist strains admit GalNAc- and Gal-modified Le^b^ (ALe^b^ and BLe^b^), whereas the specialist strains only bind to naked Le^b^ and are prevalently found in populations such as the South American Amerindians, where blood group O is highly frequent [45]. The structural and mechanistic data for *H. pylori* ABO/Le^b^ glycan binding has been recently revealed, showing that single amino acid substitutions can control the BabA binding affinity towards ABO or O histo-blood group antigens [46].

*H. pylori* infection susceptibility by BabA-positive strains has been shown to be affected by the individual secretor status [47,48,49]. The secretor phenotype is defined by the FUT2 (Se) enzymatic activity. The genetic polymorphisms in the FUT2 gene determine the capacity to produce the fucosylated type 1 antigens H-type 1 and Le^b^. In agreement, Fut2-null mice present impaired BabA-mediated binding [50].

*H. pylori* adhesion mediated by the BabA-host fucosylated antigens interaction is of high clinical relevance since infection with BabA-positive strains is associated with a poorer prognosis and increased GC risk [51].

The spectra of *H. pylori* glycan ligands in healthy gastric mucosa also includes the GalNAcβ1-4GlcNAc motif (known as *N*,*N*′-diacetyllactosediamine [lacdiNAc]). This glycan sequence is carried by the gastric MUC5AC mucin and is recognized by another member of the large Hop family of *H. pylori* OMP, the HopD, renamed lacdiNAc-binding adhesin (LabA) due to its affinity properties [52,53]. The clinical impact of the expression of *H. pylori* LabA adhesin in patient’s prognosis remains to be addressed.

#### 3.1.1. Modulation of Gastric Glycosylation Induced by *Helicobacter pylori* Infection and Inflammation

*H. pylori* infection promotes gastritis with recruitment of inflammatory cells and increased secretion of pro-inflammatory molecules. The gastric mucosa inflammation is accompanied by a shift of its glycosylation profile with de novo expression of negatively charged glycan moieties, including sialylated and sulfated antigens [54,55,56,57]. Interestingly, *H. pylori* is able to upregulate the expression of specific host glycosyltransferases, including the GlcNAc-transferase β3Gnt5, leading to biosynthesis of sialylated Lewis antigens [56,58]. These sialylated antigens, SLe^a^ and SLe^x^, are recognized by the Sialic acid binding adhesin (SabA) that promotes *H. pylori* adhesion to the inflamed gastric mucosa [54]. SabA is polymorphic in binding to sialylated antigens and different binding affinities to sialyl dimeric Le^x^ antigen, SLe^a^ and dialyllactosamine glycoconjugates are found among strains [59]. The determination of the three-dimensional structure of the SabA adhesin extracellular region has shown that its ***N***-terminal domain functions as a sugar-binding domain, with a cavity lined by conserved amino acids which likely acts as a very selective ligand-binding site [60].

The expression of the SabA adhesin is tightly regulated by different molecular mechanisms including phase variation and gene conversion [54,61,62,63,64]. The dynamic and plastic regulation of *H. pylori* OMP expression contributes for the bacteria fitness in inflamed mucosa and for its capacity to adapt to the host changes, including glycophenotype alterations.

#### 3.1.2. Novel Treatment Strategies Targeting *Helicobacter pylori* Glycan-Mediated Adhesion

The first line therapy for *H. pylori* infection consists of a combination of two antibiotics, clarithromycin and amoxicillin or metronidazole, and a proton-pump inhibitor (PPI). However, this therapeutic strategy is only effective in approximately 70% of the individuals [65]. As an alternative approach, the sequential treatment combines a period of PPI-amoxicillin with a period of PPI-clarithromycin-metronidazole (or tinidazole), although a higher efficiency is observed when the three antibiotics are taken simultaneously with a PPI (non-bismuth quadruple therapy). Bismuth-containing quadruple therapy is also considered for some patients [62,65]. The eradication success of these antibiotic-based treatments is compromised by the bacterial antibiotic resistance rates and patient compliance.

Based on the critical role that glycans play in the adhesion of *H. pylori* to the gastric epithelium and the establishment of a chronic infection, different strategies aiming the blockage of the glycan-mediated adhesion have been proposed. These alternative treatment strategies include the use of glycan-decorated chitosan microspheres, which were shown to prevent and remove *H. pylori* binding to gastric cell lines and to gastric mucosal tissue sections [66,67].

While some glycan epitopes are required for the colonization of the gastric mucosa by *H. pylori*, other glycan structures have been shown to be deleterious for the bacteria. *H. pylori* is rarely found in the deeper glands of the stomach, which show co-expression of MUC6 and terminal α1,4-GlcNAc (Figure 1aII). This is explained by the natural antibiotic effect of the α1,4-GlcNAc terminal glycan that inhibits the biosynthesis of cholesteryl-alpha-d-glucopyranoside and, therefore, compromises the bacterial wall integrity [36,68]. The α1,4-GlcNAc-capped mucin glycans are recognized by the Human Trefoil Factor 2 (TFF2) lectin, which may act as a mediator of their inhibitory activity on *H. pylori* growth [69]. Based on the bactericidal effect of terminal α1,4-linked ***N***-acetylglucosamine glycans on *H. pylori*, several treatment strategies are also being developed [70,71].

### 3.2. Glycosylation Alterations in Gastric Premalignant Conditions

*H. pylori* infection-associated chronic gastritis may evolve to IM [1,72]. IM constitutes a preneoplastic lesion characterized by a transdifferentiation of the gastric epithelium into an intestinal epithelium and is associated with increased risk of gastric carcinoma development. The loss of gastric differentiation, following genetic reprogramming of the gastric mucosa, originates foci of glands with intestinal phenotype (Figure 1b). This new epithelium harbours progressive phenotypic changes and has potential to further evolve, ultimately leading to the development of a gastric adenocarcinoma (Figure 1c) [72].

There are distinct types of IM, which have different risks of evolving in the carcinogenic pathway [4,73,74]. Regarding histomorphological features, it is possible to define two major types of IM. The complete type, or type I, is characterized by a clear loss of expression of the mucins MUC1, MUC5AC, and MUC6, which are normally present in the gastric mucosa, and aberrant expression of the intestinal MUC2 mucin in goblet cells (Figure 1bI). The incomplete type (including the type II and Type III) is characterized by the simultaneous expression of the gastric type mucins, MUC1, MUC5AC and, at lesser extent, MUC6, together with the aberrant presence of MUC2, particularly in both goblet and columnar cells (Figure 1bII) [75]. The distinction between type II and type III IM is based on the mucins produced by columnar cells. In type II IM, neutral and/or sialylated mucins are present. As for type III IM, sulfated mucins are identified [76,77]. The risk and clinical implications of preneoplastic lesions, such as IM, atrophic gastritis, and epithelial dysplasia of the stomach have been extensively discussed in the literature [78].

The expression pattern of Lewis antigens is altered in IM, namely the ectopic expression of Le^a^ in IM cells from individuals with a secretor phenotype [79,80,81]. The expression of terminal α1,4-GlcNAc glycans in IM areas has also been shown [37].

In addition, accumulation of the precursor structures T, Tn and sialyl-Tn (STn) can be detected in premalignant lesions of the gastrointestinal tract. In IM, Tn is found in columnar cells [82], whereas STn is abundantly expressed in goblet cells [83], both in the complete and incomplete types. The expression of STn is controlled by the activity of the ST6GalNAc-I enzyme [84,85]. ST6GalNAc-I has been also identified to be overexpressed in IM and its detection co-localizes, at the cellular level, with STn expression, being restricted to the perinuclear region [85]. The CDX2 homeobox transcriptor factor, normally expressed in intestinal cells, has been shown to regulate *ST6GALNAC1* gene leading to the overexpression of STn [86].

Taking into consideration that IM represents a premalignant condition of the intestinal subtype of gastric carcinoma, the search for novel biomarkers representative of this lesion is of major interest. Several alterations in the glycosylation process have already been described throughout the progression towards cancer. In fact, STn has been reported to be an independent factor of poor prognosis (reviewed in [23]). MUC2 mucin has been identified to be the major carrier of the STn truncated structure in IM and gastric carcinoma (Figure 1bI,II) [87]. Plasminogen was also found to be modified with STn in the serum of patients harbouring both GC and precursor lesions, and may represent a potential novel biomarker for non-invasive screening and diagnostic applications [88].

Gastric dysplasia is characterized by atypical changes in nuclear morphology and tissue architecture [4]. Dysplastic lesions may coexist with or in reaction to an adjacent neoplasm or can be dedifferentiated lesions with the capacity to acquire the biological characteristics of an adenocarcinoma [89]. Regarding the histochemical features of dysplastic lesions, their mucin gene expression pattern has been reported to be similar to the one observed in the incomplete type IM, where intestinal and gastric mucins are co-expressed and associated with both type 1 and type 2 Lewis antigens [34]. One of the most characteristic aberrant glycosylation features of dysplastic lesions is the increased expression of the STn antigen [82].

## 4. *O*-Glycosylation Alterations in Gastric Carcinoma

### 4.1. Aberrant Glycophenotype of Gastric Carcinoma Cells

Altered glycosylation is a hallmark of carcinogenesis with implications in the disease outcome. During the process of malignant transformation, there is the disruption of the highly-regulated normal gastric glycophenotype. Several molecular mechanisms are responsible for aberrant glycosylation modifications, leading to a variety pattern of glycosylation profiles, which result in increasing molecular heterogeneity and functional diversity within cell populations. Several factors may influence glycan biosynthesis, such as differential expression of glycosyltransferases, dysregulated function of glycosyltransferase chaperons, and relocation of the ppGalNAc-Ts that initiate *O*-glycosylation from Golgi to the endoplasmatic reticulum (reviewed in [5,6]). Because alterations of the normal glycosylation profile are already present in precancerous lesions may constitute the early steps of the carcinogenesis cascade, the aberrant glycan signatures of GC are highly heterogeneous and significantly different depending on tumour subtype [90].

One of the major molecular events taking place during gastric carcinogenesis is the loss of the well-defined mucin expression pattern observed in the normal epithelium [91]. In intestinal type tumours, cancer cells exhibit an aberrant mucin expression pattern characterized by a decrease in the levels of the gastric mucins MUC5AC and MUC6 and the concomitant upregulation of genes codifying for intestinal mucins, including the de novo expression of MUC2, as well as abnormally elevated levels of expression of MUC3, MUC4 and MUC5B [11,13,14,90,92,93,94]. Differently from other gastric mucins, MUC1 has been classified as an oncoprotein and its reported overexpression in GC is associated with the dismal prognosis of patients [95]. MUC1 is able to modulate the Wnt signalling pathway through the formation of an intracellular complex with β-catenin, which is, in turn, capable of co-activating cyclin-D1 expression in the cell nucleus, ultimately promoting tumorigenesis by allowing cancer cells to avoid apoptotic pathways [96]. Besides its altered expression in GC cells, MUC1 aberrant glycosylation has also been well documented (Figure 1cI) [15]. The deficient extension of its *O*-glycan side chains leads to the production of underglycosylated forms of MUC1 where the exposure of immunogenic epitopes can trigger the immune response and cancer-associated inflammation [97]. In addition to altered expression levels and aberrant glycophenotypes, genetic polymorphisms affecting gastric mucins further influence the risk of GC development [98,99,100].

Alterations in the expression and activity of several glycosyltransferases constitute one of the major molecular mechanism underlying the expression of aberrant immature carbohydrate structures in mucins and have provided valuable information on GC cell behaviour [101]. A recent study uncovered the prognostic value of markedly reduced levels of ppGalNAc-T5 in patients harbouring GC [28]. On the other hand, ppGalNAc-T10 overexpression in the gastric malignant mucosa was associated with the degree of tumour differentiation [30]. Another study showed that increased levels of ppGalNAc-T6 in a set of gastric carcinoma specimens positively correlated with venous invasion [29]. Additionally, the abnormally increased expression of ppGalNAc-T3 in GC has been associated with tumour differentiation degree and ability to metastasize [102]. Several in vitro studies performed on GC cell lines further support the crucial role played by the dysregulation of ppGalNaC-Ts expression and activity in the malignant phenotype of GC cells [103,104,105].

Due to a premature stop of the extension of *O*-glycosylation, intestinal type GC often overexpresses immature and truncated *O*-glycans, such as Tn, STn, T and sialyl-T (ST) antigens (Figure 1cI) [83,106,107]. STn overexpression in GC has a major impact in tumour cell behaviour and aggressiveness [108]. In most gastric carcinoma cases, ST6GalNAc-I is found to be co-expressed with the STn structure [85].

In gastric neoplastic cells, the loss of the highly co-regulated expression of specific fucosyltransferases and their distinct mucin substrates further contributes to the biosynthesis of tumour-associated antigens. This observation is supported by studies performed on a set of gastric carcinomas where the simultaneous non-specific expression of *FUT1* and *FUT2* transcripts with both MUC5AC and MUC6 was observed [34,92]. A different study showed increased levels of FUT4 mRNA in comparison to the normal gastric mucosa. Alterations in the levels of fucosyltransferase enzymes occurring during gastric carcinogenesis consequently lead to the aberrant expression of their carbohydrate products, the Lewis blood group antigens, regardless of the individual phenotype. Thus, the reported increased levels of Le^a^ in both GC cell lines and tissues is due to the concomitant upregulation of *FUT3* gene [109,110]. As opposed to Le^a^, the levels of the remaining neutral Lewis antigens show a progressive reduction following neoplastic transformation of the gastric epithelium [33,111]. 

The overexpression of the sialylated Le^a^ and Le^x^ is another well-established event during gastric carcinogenesis [112,113,114,115]. These sialylated structures are ligands for E- and P-selectins found in endothelial cells, allowing the malignant cells binding to the endothelium (Figure 1c(II)) [116,117]. The elevated levels of these terminal carbohydrate structures have been extensively associated with a more aggressive tumour behaviour due to an increased invasive and metastatic potential [113,118,119,120,121]. The upregulation of sialylated Lewis antigens is, in turn, caused by the marked abnormally increased levels of the glycosyltransferases responsible for their biosynthesis. In fact, the overexpression of ST3Gal3, ST3Gal4 and FUT5 in gastric tumour cells was demonstrated in both in vitro and in vivo studies [88,115,122,123]. Moreover, it was demonstrated that the overexpression of sialyltransferase ST6Gal1 in tissues of GC patients [115,122]. The enzyme is responsible for the formation of the sialylated CDw75 antigen, which has been previously associated with tumour aggressiveness [124].

Defining the *O*-glycoproteome of gastric tumour cells would allow the identification of the proteins carriers of the altered glycans, allowing the study of their role in carcinogenesis and their disclosure as a set of putative biomarkers with potential clinical applications. However, due to the complexity and heterogeneity of these glycan structures, this task remains a major challenge in the field. The recent implementation of genome editing tools combined with high-throughput mass spectrometry technology has allowed the characterization of the *O*-glycoproteome of GC cells, leading to the identification of novel biomarkers [125,126].

### 4.2. Crosstalk between Altered Glycosylation and Tyrosine Kinase Receptor Activation in Gastric Cancer

There is a limited number of molecular markers available for evaluation of gastric lesions as well as detection and prognostic evaluation of GC. Since dysregulation of tyrosine kinases receptors (TKRs) activity has been shown to actively contribute to gastric oncogenesis and disease progression, TKRs are recognized as appealing therapeutic targets.

Alterations in distinct TKRs (including c-Met, EGFR, HER2, and RON) have been associated with GC progression [127,128,129,130,131,132]. In addition, activation of several signalling pathways due to abnormal TKR activity (Figure 1cI) has also been implicated in the modulation of distinct aspects of tumour cell behaviour, namely proliferation, migration, angiogenesis and invasion, and associated with the clinical outcome of GC patients [133,134].

Many studies correlate altered cell glycosylation of cancer cells to the activation of important signalling pathways [6,135]. Recently, it has been demonstrated that the induced expression of cancer associated antigen SLe^x^ in GC cells, through the overexpression of the ST3GalIV enzyme, leads to an increased activation of c-Met receptor and its downstream signalling axis, ultimately conferring cancer cells a higher invasive capacity [136]. The c-Met receptor, also known as hepatocyte growth factor receptor (HGFR), is normally expressed in a variety of epithelial and endothelial cells and its aberrant expression and activation have been associated with proliferation, migration and invasion of GC cells [137,138]. In addition to its role in gastric carcinoma, c-Met overexpression has also been described in gastric precancerous lesions such as IM and dysplasia [139].

The mechanisms leading to increased c-Met expression and activation during gastric carcinogenesis have not yet been completely understood. It was reported that *H. pylori* infection leads to the activation of c-Met in gastric epithelial cells with the concomitant activation of a signalling cascade contributing to tumour progression [140]. Furthermore, *H. pylori* virulence factors modulate c-Met-associated PI3K-AKT signalling axis, ultimately contributing to cancer cell malignant behaviour [140,141,142,143,144].

CD44 constitutes a cell surface adhesion molecule and the receptor for the hyaluronan glycan, which is expressed by a variety of cells including the ones of the gastric epithelium, and may be implicated in gastric carcinogenesis. Moreover, it has been recently identified as a GC stem cell marker [145]. In addition, several studies have identified the CD44 receptor containing the variant exon 6 as an essential co-activator of c-Met [146]. Furthermore, this association was recently shown to occur in response to *H. pylori* infection [147]. As described above, *H. pylori* has the capacity to adhere to the gastric mucosa via interaction with host glycan epitopes. During infection, *H. pylori* is able to actively modulate the gastric epithelium glycophenotype by inducing the expression of new glycan epitopes that serve as bacterial adhesion sites and the activation of several signalling pathways. It has been suggested that *H. pylori* infection is responsible for the upregulation of CD44, either directly or by induced local inflammatory response [148].

Apart from the direct role played by CD44 activation in cancer, the expression of additional specific variants as v6 and v3 has also been reported in GC. In fact, expression of CD44 variant v6 was associated with invasive intramucosal gastric carcinoma and identified as a marker of premalignant lesions like IM and dysplasia [149]. Although the expression of CD44 variant v6 constitutes a molecular hallmark of several human cancers, the exact mechanisms underlying its contribution to tumorigenesis remain unclear. The structural and functional diversity of CD44 variants originated by alternative splicing mechanisms and differential glycosylation, significantly influence the receptor’s role in pro-inflammatory events such as cell-cell and cell-matrix interactions, which constitute critical processes during carcinogenesis [150,151,152,153].

As the principal ligand of CD44, hyaluronan acts as an important player in the modulation of intracellular signalling pathways [154,155]. Furthermore, it has been described that hyaluronan-mediated CD44 activation requires posttranslational modifications such as glycosylation of the receptor’s extracellular domain and/or the phosphorylation of specific Serine residues in its cytoplasmic tail, allowing the receptor to transit from an inactive (low-affinity) to an active (high-affinity) state [150]. Interestingly, CD44 glycoforms containing STn have been identified in the serum of GC patients [125].

Moreover, it has been recently demonstrated that the activation of other TKRs can be modulated by altered glycosylation in gastric carcinoma cells. The induction of SLe^x^ expression in GC cells by the overexpression of a single enzyme, ST3GalIV, leads to a drastic remodelling of cancer cell glycophenotype, with the concomitant activation of different TKRs, such as RON, ultimately tuning cancer cell behaviour [132,136,156,157].

## 5. Conclusions

Mucin glycosylation plays a crucial role in various aspects of gastric diseases. The major role of mucins and their abundant *O*-glycans are crucial in *H. pylori* adhesion and chronic infection of the gastric mucosa. Furthermore, alterations of glycosylation, occurring in preneoplastic lesions and in gastric carcinoma, have been shown to be involved in several molecular processes underlying the gastric carcinogenesis, as well as in the different steps of cancer progression. Finally, it is envisioned that these mucin *O*-glycosylation alterations have potential applications in the clinical setting, including screening, prognosis of the patient and as indicators of response to therapy.

Comprehension of specific glycosylation alterations that occur during the precancerous cascade and tumour progression is crucial for both researchers and clinicians. Indeed, glycosylated epitopes have been already established as powerful biomarkers in cancer diagnosis. Finally, glycan-based therapies and the role of glycosylation in patients’ response to therapy may represent important tools in the future clinical management of GC patients.

## Abbreviations

The following abbreviations are used in this manuscript:

MUC	Mucin
ppGalNAc-T	UDP-GalNAc:polypeptide *N*-Acetylgalactosaminyltransferase
SLe^a^	Sialyl-Lewis^a^
SLe^x^	Sialyl-Lewis^x^
FUT	Fucosyltransferase
*H. pylori*	*Helicobacter pylori*
GC	Gastric Cancer
OMP	Outer Membrane Proteins
BabA	Blood Group Antigen Binding Adhesin
LabA	LacdiNAc-Binding Adhesin
SabA	Sialic Acid Binding Adhesin
PPI	Proton-Pump Inhibitor
IM	Intestinal Metaplasia
STn	Sialyl-Tn
ST	Sialyl-T
TKR	Tyrosine Kinase Receptor
